# Accuracy, Repeatability, and Reproducibility of a Hand-Held Structured-Light 3D Scanner across Multi-Site Settings in Lower Limb Prosthetics

**DOI:** 10.3390/s24072350

**Published:** 2024-04-07

**Authors:** Andrea Giovanni Cutti, Maria Grazia Santi, Andrew H. Hansen, Stefania Fatone

**Affiliations:** 1 INAIL, Via Rabuina 14, Vigorso di Budrio, 40054 Bologna, Italy; ag.cutti@inail.it; 2 Department of Industrial Engineering, University of Padova, 35131 Padova, Italy; mariagrazia.santi@phd.unipd.it; 3 Minneapolis VA Health Care System, One Veterans Drive, Minneapolis, MN 55417, USA; andrew.hansen2@va.gov; 4 Department of Family Medicine and Community Health, Department of Biomedical Engineering, University of Minnesota, Minneapolis, MN 55455, USA; 5 NUOPC, Department of Physical Medicine and Rehabilitation, Northwestern University, 680 N Lake Shore Dr, Suite 1100, Chicago, IL 60611, USA; 6 Department of Rehabilitation Medicine, University of Washington, 1959 NE Pacific St., P.O. Box 356490, Seattle, WA 98195, USA

**Keywords:** structured-light 3D scanner, hand-held 3D scanner, accuracy, repeatability, reproducibility, amputation, limb loss, prosthetics

## Abstract

The aim of this work was to assess the accuracy, repeatability, and reproducibility of a hand-held, structured-light 3D scanner (EINScan Pro 2X Plus with High Definition Prime Pack, SHINING 3D Tech. Co., Ltd., Hangzhou, China), to support its potential use in multi-site settings on lower limb prosthetics. Four limb models with different shapes were fabricated and scanned with a metrological 3D scanner (EINScan Laser FreeScan 5X, SHINING 3D Tech. Co., Ltd., Hangzhou, China) by a professional operator (OP0). Limb models were then mailed to three sites where two operators (OP1, OP2) scanned them using their own structured-light 3D scanner (same model). OP1 scanned limb models twice (OP1-A, OP1-B). OP0, OP1-A, and OP2 scans were compared for accuracy, OP1-A and OP1-B for repeatability, and OP1-A and OP2 for reproducibility. Among all comparisons, the mean radial error was <0.25 mm, mean angular error was <4°, and root mean square error of the radial distance was <1 mm. Moreover, limits of agreement were <3.5% for perimeters and volumes. By comparing these results with respect to clinically-relevant thresholds and to the literature available on other 3D scanners, we conclude that the EINScan Pro 2X Plus 3D Scanner with High Definition Prime Pack has good accuracy, repeatability, and reproducibility, supporting its use in multi-site settings.

## 1. Introduction

Hand-held digital 3D scanners are used increasingly in prosthetics and orthotics in both clinical practice and research. In clinical practice, hand-held 3D scanners are used to digitize certain steps in the process of fabricating prosthetic and orthotic devices, primarily assisting with capturing the shape of the relevant body part or their negative or positive casts. This scanned information may also be useful for research purposes, as investigators explore new methods for improving the person-device interface, e.g., to customize and fit soft orthoses [1] or prosthetic sockets, or for studying how the shape of sockets is obtained, starting from the limb shape or shape of the negative cast, passing through the unrectified positive mold, the rectified positive mold, the check (diagnostic) socket, and the final definitive socket. This process is called quantitative shape analysis, and, when applied to a pool of prosthesis users treated by one or more prosthetists, can support repeatability/reproducibility analyses and the construction of templates with average rectification maps [2,3,4,5,6,7,8].

Given the promise that digitization has for improving various aspects of prosthetic practice [9,10,11], there has been a growing interest in assessing 3D scanner performance. For example, Seminati et al. compared the performance of the Artec EVA 3D scanner (Artec3D, Niederanven, Luxembourg) to a high precision Romer Laser 3D Scanner (Hexagon, London, UK) [12] and in a subsequent publication, to the Omega 3D Scanner (WillowWood, Mt Sterling, OH, USA) [13]. Dickinson et al. compared the performance of the VIUScan (Creaform Inc., Lévis, QC, Canada), Go!SCAN (Creaform, Inc., Lévis, QC, Canada), and Sense 3D (3D Systems, Rock Hill, SC, USA) [14] and later, the Omega Sense (3D Systems, Rock Hill, SC, USA) and iSense 3D Scanners (3D Systems, Rock Hill, SC, USA) [15]. Kofman et al. compared the performance of four 3D scanners: TT Design (Otto Bock, Duderstadt, Germany), Omega (WillowWood, Mt Sterling, OH, USA), Biosculptor (Biosculptor, Irvine, CA, USA) and Rodin4D (Rodin4D, Mérignac, France) [16]; and in a later publication, three 3D scanners: Rodin4D (Rodin4D, Mérignac, France), Omega Tracer (Biosculptor, Irvine, CA, USA) and Omega Biosculptor (Biosculptor, Irvine, CA, USA) [17]. Hernandez & Lemaire [18] evaluated the ability of a Galaxy S5 smartphone (Samsung, Suwon, Republic of Korea) to scan the interior of sockets.

In general, these studies described 3D scanner performance based on outcomes that can be classified in two categories: (1) “global” and (2) “Orthotics and Prosthetics-(O&P-) relevant”. Global outcomes describe differences between scanned surfaces as a whole, for instance, the relative distance between closest points. For example, Sanders et al. [19] and Seminati et al. [12] reported that radial errors greater than the thickness of two socks of 1 ply, i.e., between 1.5 and 1.8 mm, produce clinically-relevant differences in gait, quality of fit, comfort, and user satisfaction. Seminati et al. [12] reported other acceptable 3D scanners for use in prosthetics to have a root mean square error of the radial distance (RMSE-RE) < 1 mm. In comparison, O&P-relevant outcomes encompass parameters typically used in clinical practice, e.g., differences in perimeters of cross sections and volumes. Mehmood et al. [20] and Dickinson et al. [15] established that a minimal detectable change (MDC) below 3.5%, both for perimeter and volume, is well within acceptable thresholds. A 1% bias for perimeter and volume was considered acceptable by Seminati et al. [12]. 

Among currently available 3D scanner assessments, no prior evaluations have accounted for a scenario where multiple physically distant sites are involved in data collection, using the same 3D scanner model but different staff. Therefore, the effect of inconsistencies between 3D scanner units, environmental conditions of use, staff skills, technological competence, and training on scanning have not been assessed. The assessment of accuracy (error relative to a gold standard), repeatability (variation in repeated measures made on the same subject under identical conditions, e.g., intra-operator), and reproducibility (variation in measurement made on the same subject under changing conditions, e.g., inter-operator) [21] in these contexts is an essential requirement before merging data collected by prosthetic facilities with multiple offices or in multi-site studies. This is, for instance, the case for a multi-center clinical trial conducted by our research centers (https://clinicaltrials.gov/study/NCT04141748 accessed on 26 March 2024) aimed at comparing different casting techniques used to prepare a socket for persons with lower limb amputation, in terms of shape consistency, timing, and comfort. All three participating sites were equipped with the same 3D scanner (EINScan Pro 2X Plus 3D Scanner with High Definition Prime Pack, SHINING 3D Tech. Co., Ltd., Hangzhou, China), physical limb models, and provided the same instructions. The merging of data collected on participants enrolled across the three sites is predicated upon the accuracy, repeatability and reproducibility of the 3D scanner when used by operators at each site under their unique environmental conditions.

Finally, no data are available for the EINScan Pro 2X Plus 3D Scanner (SHINING 3D Tech. Co., Ltd., Hangzhou, China) with high-definition (HD) supplementary add-on camera (in short, a structured-light 3D scanner). Compared to other devices, this structured-light 3D scanner may offer a level of accuracy that would be particularly beneficial for quantitative shape analysis, with a fast acquisition time even for operators with limited experience and with an accessible purchase cost. Hence, the aim of this study was to test, in a multi-site setting, the accuracy, repeatability, and reproducibility of the EINScan structured-light 3D scanner. 

## 2. Materials and Methods

### 2.1. 3D Scanners

For this study, we assessed the EINScan Pro 2X Plus with added HD Prime Pack (SHINING 3D Tech. Co., Ltd., Hangzhou, China): an external add-on camera mechanically locked to the 3D scanner with a latch-on USB port that allows for HD scanning. To ensure the best performance, at the beginning of each measurement session the 3D scanner was calibrated following the manufacturer’s instructions and using the etched glass board provided with the 3D scanner. This calibration required about 2 minutes to complete. All scans were completed in hand-held HD scan mode, without markers (“features mode”), setting a resolution of 0.5 mm, at 20 frames per second, with 1 million points collected per second. The manufacturer reports a volumetric accuracy in the Association of German Engineers and Association for Electrical, Electronic, and Information Technologies (VDI/VDE) 2634 Standards [22] of less than 0.05 mm, irrespective of the orientation of a ceramic calibration dumbbell.

To measure the accuracy of the EINScan Pro 2X Plus 3D Scanner with HD Prime Pack, a metrological 3D scanner was used as reference and assumed as ground truth. For this purpose, we selected the EINScan Laser FreeScan X5 (SHINING 3D Tech. Co., Ltd., Hangzhou, China), with a certified volumetric accuracy in the VDI/VDE 2634 standards of 0.03 mm [22]. 

### 2.2. Limb Models 

**Figure 1:**
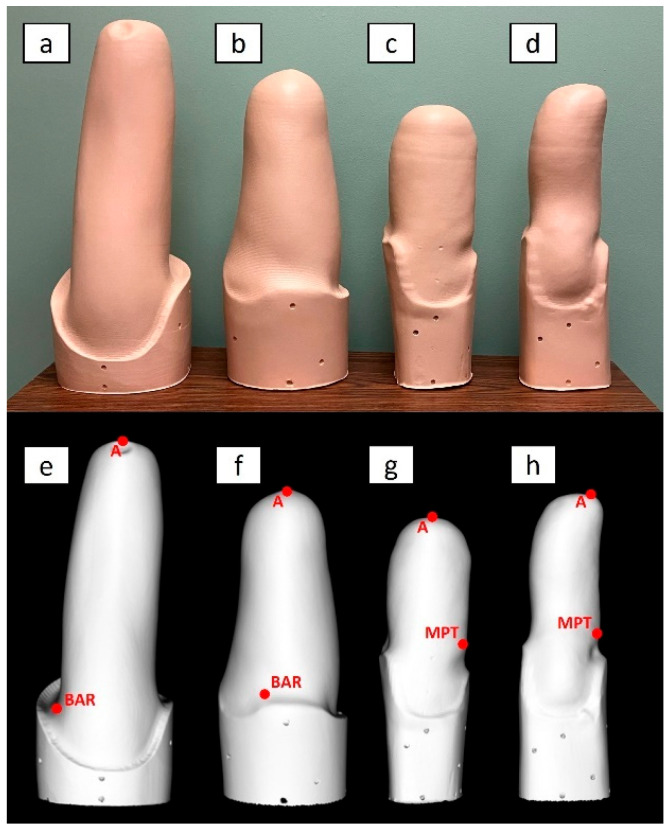
Side view of the four physical limb models (**a**–**d**) and an example of their corresponding scan (**e**–**h**). A: Transfemoral (TF) Ischial (right limb, lateral view). B: TF Aqua (left limb, medial view). C: Transtibial (TT) Cylindrical (left limb, lateral view). D: TT Conical (right limb, medial view). In the figures (**e**–**h**), the positions of anatomical landmarks are reported on the scanned shapes.

From a digital database of over 1000 sockets from one of the clinical sites, we selected four shapes (Figure 1) considered representative of a:

- Transfemoral socket for a long residual limb, with ischial containment producing undercuts/indentations around the proximal brim, referred to as “TF Ischial”;
- Transfemoral socket for a long residual limb, with ischial containment but no undercuts/indentations around the proximal brim. Since this socket was based on hydrostatic casting with the Symphonie Aqua System (Romedis GmbH, Neubeuern, Germany) [23], it will be referred to as “TF Aqua”;
- Transtibial socket for a mid-length residual limb, with a total surface bearing design and a cylindrical shape, referred to as “TT Cylindrical”;
- Transtibial socket for a long residual limb, with patellar tendon bearing design and a conical shape, referred to as “TT Conical”.

All investigators, including prosthetists, agreed that the four shapes were representative of very different socket/limb presentations. Once selected, the four limb models were carved from polyurethane foam by a robot (Ortis, Fabrica Machinale, Caschina, PI, Italy). This material was chosen because it is lightweight and less fragile than plaster casts and less likely to be damaged during shipping to different sites. To prevent the limb models from deteriorating due to impact or abrasion, they were covered in polyethylene terephthalate glycol (PETG) by vacuum forming, starting from a 0.12 mm sheet. The PETG surface was then sanded and sprayed with matte paint. Finally, 7 mm holes were drilled in the extension above the proximal socket trim lines to serve as technical landmarks to align (i.e., spatially register) the scanned shapes during data analysis.

### 2.3. Testing Sites and Operators

Three sites participated in the assessments, two in the United States (referred to herein as USA1 and USA2) and one in Europe (EU). Each site had its own structured-light 3D scanner (same model: EINScan Pro 2X Plus 3D Scanner with added HD Prime Pack). Two individuals participated in scanning at each site: Operator 1 (OP1) with experience using the 3D scanner, and Operator 2 (OP2) with minimal prior experience using the 3D scanner. The same four limb models were mailed to each site. 

**Figure 2:**
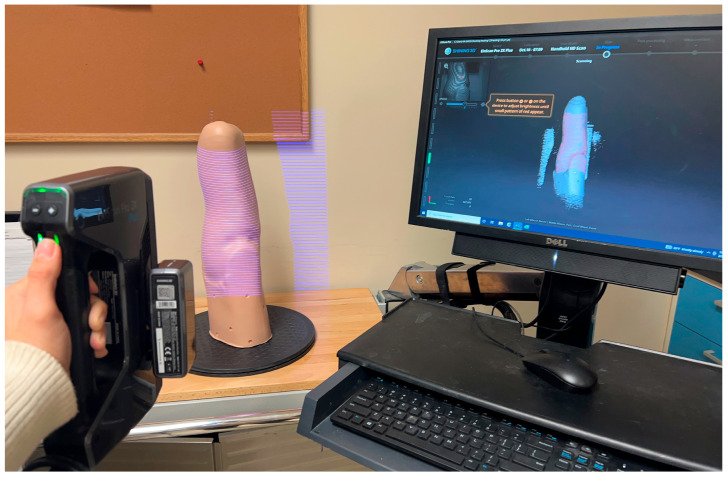
Example of scanning set-up and procedure to digitally scan one of the four residual limb models with the EINScan Pro 2X Plus 3D Scanner with added HD Prime Pack.

At each site, OP1 scanned the four limb models twice (OP1-A and OP1-B) with the structured-light 3D scanner, and OP2 scanned the four limb models once with the same structured-light 3D scanner, for a total of 12 scans per site. At the European site, an additional reference scan of each positive model (4 scans) was conducted by an independent professional Operator (OP0) using the metrological 3D scanner (EINScan Laser FreeScan 5X). All scan files are available on Dryad [24]. An example of the scanning set-up and procedure is illustrated in Figure 2.

### 2.4. Scan Processing 

**Figure 3:**
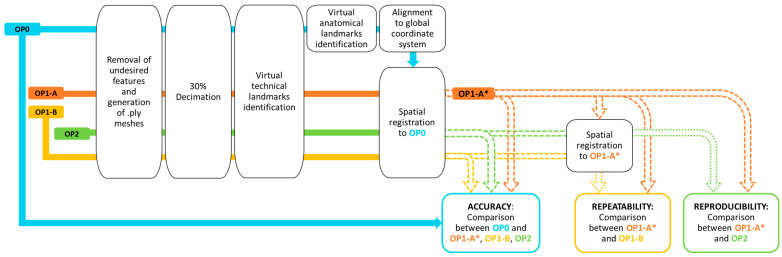
Schematics of the data processing for the scans of the four limb models collected at the three sites by operators OP0, OP1 (A and B), and OP2. The aligned scans of OP1-A on OP0 were referred to as OP1-A*.

For each model, scans were processed as summarized in Figure 3. First, the EINScan software (ExScan Pro_v3.4.0.4) was used to remove background surfaces and geometries and to generate watertight meshes in the STL file format. Then, the obtained digital meshes were processed using custom Python software [25] that takes advantage of MeshLab (MeshLab_64bit_fp v2020.07) [26] and Visual Toolkit (VTK) libraries (version 9.0.0) [27] as computational geometry engines, with Qt (PyQt5 version 5.14.2) [28] to support the graphical user interface. 

To decrease computational costs, all ten scans from each limb model were decimated to 30%, i.e., removing 70% of mesh vertices, to obtain meshes of reduced dimension in PLY file format. Then, the center of the holes on the proximal extension above the socket trim lines were identified on the scans of the limb models by an operator. These points will be referred to as technical landmarks. The next two steps involved only the mesh for OP0. Specifically, the location of the anatomical landmarks (reported in Figure 1) were manually identified on the scans of the limb models by agreement of two experienced prosthetists who were familiar with the limb models. As shown in Figure 1, the designated anatomical landmarks were the most distal point of the models (A) and, for transtibial limb models, the Mid Patellar Tendon (MPT) and, for transfemoral limb models, the midpoint Between the origin of Adductor longus and the ischial Ramus (BAR).

The two prosthetists also provided agreement regarding the alignment of the limb models in the transverse, frontal, and sagittal planes of the global coordinate system, with the origin at the most distal anatomical point of the socket (landmark A, Figure 1).

At each site, to assess the 3D scanner [21]:

(1) Accuracy: scans from OP1-A, OP1-B, and OP2 (measured meshes) were spatially registered and compared to OP0 (reference mesh); the aligned scans of OP1-A on OP0 were referred to as OP1-A*.
(2) Repeatability: scans from OP1-B (measured mesh) were spatially registered and compared to OP1-A* (reference mesh).
(3) Reproducibility: scans from OP2 (measured mesh) were spatially registered and compared to OP1-A* (reference mesh).

**Figure 4:**
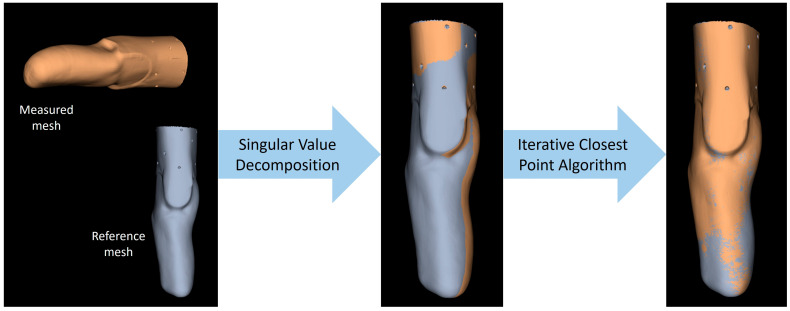
Schematic representation of the process necessary to perform spatial registration of the measured mesh with respect to the reference mesh.

Spatial registration exploited two algorithms in sequence (Figure 4). First, a rigid registration based on singular value decomposition was completed using the technical landmarks common to all scans. Then, the registration was improved with the Iterative Closest Point algorithm applied to 2500 vertices. 

### 2.5. Outcome Measures and Their Interpretation

To compare scans, two sets of outcome measures were extracted, similarly to Sanders et al. [19], Seminati et al. [12], and Dickinson et al. [15], which will be referred to as global metrics and O&P-relevant metrics. The first set, global metrics aimed to provide a global understanding of the deviations between scans, using the overall mesh of each scan. The second set, O&P-relevant metrics replicated measurements that are commonly considered to be clinically important when fitting a socket on a patient.

### 2.6. Global Metrics

**Figure 5:**
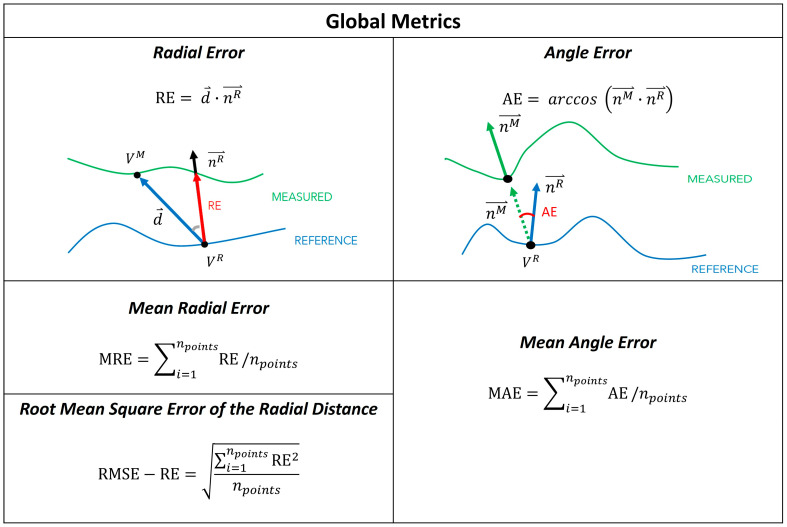
Summary of the Global Metrics and their visual explanation. Radial error (RE); point on the reference mesh (*V^R^*); distance between meshes ($\vec{d}$); point on the measured mesh (*V^M^*); mean radial error (MRE); mesh points ($n_{points}$); root mean square error of the radial distance (RMSE-RE); mean angle error (MAE); angle error (AE); normal vector to the reference mesh ($\vec{n^{R}}$); normal vector to the measured mesh ($\vec{n^{M}}$).

As part of the global metrics, we computed (Figure 5):

1. The mean radial error (MRE) [19], which provides a measure of the distance between the reference and measured mesh. For each point in the reference mesh ($V^{R}$), the radial error (RE) is defined as the signed distance (in mm) to its closest point on the measured mesh ($V^{M}$). The MRE is the average of the radial error over the whole set of reference mesh points ($n_{points}$), holes excluded. This measure has also been referred to as the scan surface height by Dickinson et al. [15].
2. The root mean square error of the radial distance (RMSE-RE) was also computed for comparison with Seminati et al. [12].
3. The mean angle error (MAE), which provides a measure of the shape differences between the reference and measured mesh [19]. The angle error (AE) is the deviation of the normal unit vectors at closest points, measured in degrees. In detail, let us assume that $\vec{n^{R}}$ is the normal to the reference mesh at its point $V^{R}$, and $\vec{n^{M}}$ is the normal vector to the measured mesh at the closest point to $V^{R}$ on that mesh. The angle error is the angular deviation of $\vec{n^{R}}$ and $\vec{n^{M}}$. MAE is the average of the angular error over the whole set of points of the reference mesh ($n_{points}$), holes excluded.

Based on the clinical evidence collected by Sanders et al. [19], a transtibial socket is considered clinically unacceptable when, first, the MRE is greater than 0.25 mm and, second, MAE is greater than 4° with respect to a well-fitted socket. We expected 3D scanner errors for the transtibial limb models to be less than these minimal clinically important differences (MCIDs). Since specific data on transfemoral sockets are lacking, we conservatively assumed the same acceptability thresholds for 3D scanner errors for transfemoral limb models.

Based on results from Sanders et al. [19] and Seminati et al. [12], radial errors greater than the thickness of two socks of 1 ply, i.e., between 1.5 and 1.8 mm, produce clinically-relevant differences in gait, quality of fit, comfort, and user satisfaction. Moreover, Seminati et al. [12] reported other acceptable 3D scanners for use in prosthetics to have RMSE-RE < 1 mm. Hence, we set our acceptability threshold to RMSE-RE < 1 mm. 

### 2.7. O&P-Relevant Metrics

**Figure 6:**
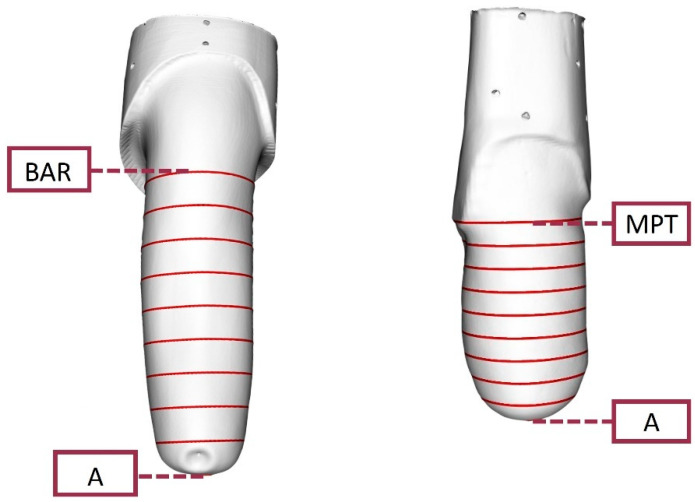
Example of the nine cross sections (indicated by red horizontal lines) from A to BAR for the Transfemoral (TF) Ischial limb model and from A to MPT for the Transtibial (TT) Conical limb model.

It is common for prosthetists to consider cross-sectional perimeters (sometimes also referred to as circumferences, although this term is not accurate for objects that are not circles) at various heights along the longitudinal axis of the residual limb (or positive model), as a primary guide to check for changes in residual limb volume and shape, for model rectification and socket verification [29]. For this reason, we computed nine, equally spaced cross sections (Figure 6) that for:

1. transtibial models, ran between landmark A and the Mid Patellar Tendon (MPT);
2. transfemoral models, ran between landmark A and the midpoint Between the origin of Adductor longus and the ischial Ramus (BAR).

Another clinically relevant outcome that has been extensively reported in the literature is the residual limb volume [30]. Since this outcome measure was also reported in previous studies evaluating 3D scanner accuracy, repeatability, or reproducibility [12,15], we calculated the volume enclosed in the scans from landmark A to the most proximal cross section. 

Consistent with recommendations from agreement studies [21,31] and previous evaluations of 3D scanners [12,15,16,19], a Bland–Altman plot was used to assess measurement bias and the MDC (also referred to as CR, the coefficient of repeatability) between scans. The MDC sets the level of disagreement between measures: if the disagreement is too large, measures cannot be considered equivalent and clinically meaningful information might be lost. Since models have very different perimeters and volumes, and to facilitate comparison, differences between measures were reported as percent differences from the reference scan (OP0 or OP1-A*). Moreover, to ease interpretation of perimeters collected at different heights along a limb model, the percentage height of the cross section was reported on the Y axis of the plot, to resemble the vertical orientation of a limb model instead of the X axis as is typical for Bland–Altman plots. 

Results about bias and MDC cannot be interpreted with a statistical test [31,32]. It is up to the research team or healthcare professional to decide what is an acceptable level of uncertainty to avoid a wrong diagnosis, treatment, socket design, or technical misinterpretation. Based on clinical evidence collected by Lilja and Öberg [33], Sanders et al. [34], and Lilja et al. [35] about clinically appreciable differences in gait, quality of fit, comfort, satisfaction, and muscle activation, as a function of socket perimeter and volume differences compared to a well fit socket, Mehmood et al. [20] and Dickinson et al. [15] established that a MDC below 3.5%, both for perimeter and volume, is well within acceptable thresholds. A 1% bias for perimeter and volume was considered acceptable by Seminati et al. [12]. Therefore, for this study we considered acceptable limits of agreement (calculated as bias ± MDC) of 3.5%.

## 3. Results

All sites reported that no more than 5 minutes were required to scan the object and obtain the STL mesh. The height and volume of the limb models is reported in Table 1, as measured by OP0.

### 3.1. Global Metrics

**Figure 7:**
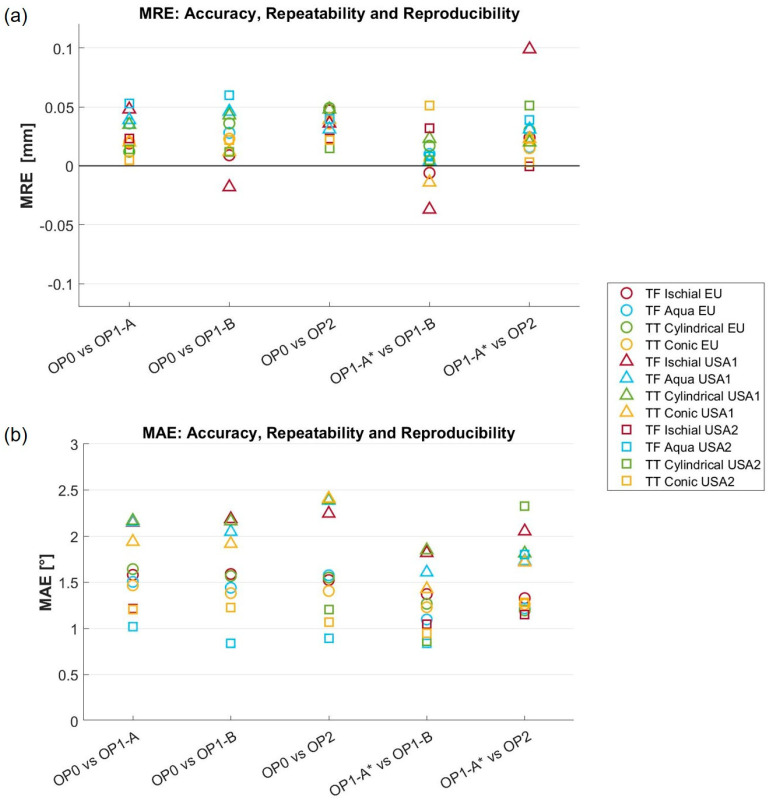
Results for the (**a**) mean radial error (MRE) and (**b**) mean angle error (MAE) across all four limb models and three testing sites (USA1, USA2, and EU). Accuracy: OP0 vs. OP1-A, OP0 vs. OP1-B, OP0 vs. OP2. Repeatability: OP1-A* vs. OP1-B. Reproducibility: OP1-A* vs. OP2. The aligned scans of OP1-A on OP0 were referred to as OP1-A*.

Results for global metrics are reported in Figure 7, Table 2, and the Table S1 Dataset. Specifically, Figure 7 reports results for the MRE and MAE regarding accuracy, repeatability, and reproducibility, across all models and testing sites. The MRE, with a worst-case value of 0.1 mm, is well below the 0.25 mm considered an acceptable threshold. With the exclusion of two values (TF Aqua, USA2, accuracy; TF Ischial, USA1, reproducibility), the range of variation is from −0.05 mm to 0.05 mm across accuracy, repeatability, and reproducibility. Similarly, for the MAE, the range of variation is between 1° to 2.5° for accuracy, repeatability, reproducibility, models, and sites. This range is appreciably lower than the 4° considered an acceptable threshold.

Table 2 reports the range for the RMSE-RE. For accuracy across models and sites, the results are between 0.06 and 0.27 mm, for repeatability between 0.04 and 0.16 mm, and for reproducibility between 0.04 and 0.52 mm. All values are about half the 1 mm considered an acceptable threshold.

### 3.2. O&P-Relevant Metrics

**Figure 8:**
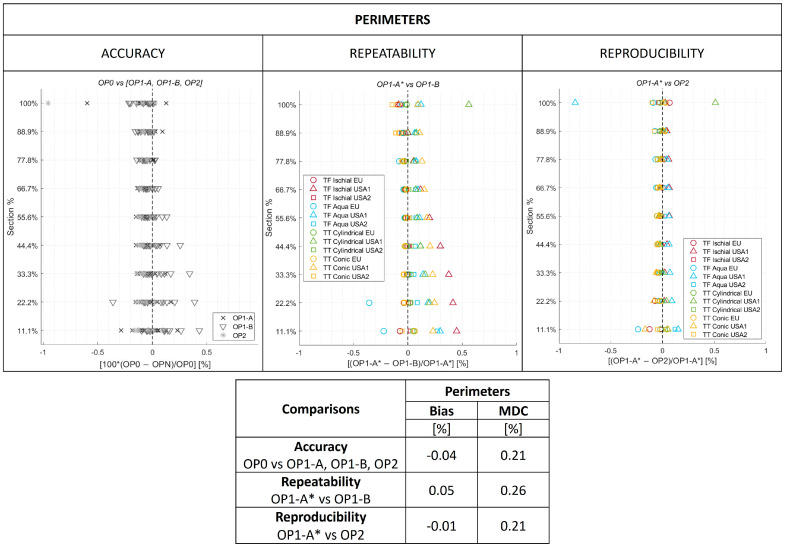
Modified Bland–Altman plots for accuracy, repeatability, and reproducibility of perimeters. Overall bias and minimal detectable change (MDC) are reported in the embedded table. The aligned scans of OP1-A on OP0 were referred to as OP1-A*. OPN represents OP1-A, OP1-B and OP2.

**Figure 9:**
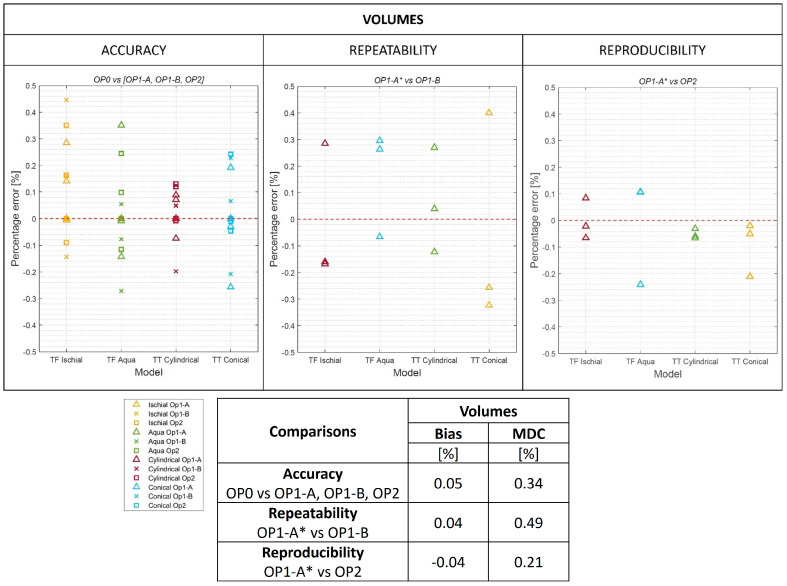
Modified Bland–Altman plots for accuracy, repeatability, and reproducibility of volumes. Overall bias and minimal detectable change (MDC) are reported in the embedded table. The aligned scans of OP1-A on OP0 were referred to as OP1-A*.

Results for the O&P-relevant metrics are reported in Figure 8 for perimeters, Figure 9 for volumes, and the S1 Dataset. 

In Figure 8, for accuracy, symbols refer to the comparison between OP0 and OP1-A, OP1-B and OP2, respectively. For each cross section and comparison, 12 identical symbols are reported (four models per three testing sites—USA1, USA2, and EU). For repeatability and reproducibility, symbols allow identification at each cross section of each model and testing site. Overall bias and MDC for accuracy, repeatability, and reproducibility are reported in the embedded table.

For perimeter results, bias ranged between −0.04% and 0.05%, and MDC ranged between 0.21% and 0.26%. Overall, limits of agreement spanned between −0.25% to 0.31%, which is below the 3.5% considered an acceptable threshold. Plots in Figure 7 show a few outliers at the 100% cross section only for TT Cylindrical and TF Aqua limb models scanned at one site, however even these outliers are far below the acceptable threshold. 

In Figure 9, differences were calculated for the volume enclosed by the 100% cross section, i.e., between A and MPT for transtibial models, and between A and BAR for transfemoral models. Also, in this case, overall bias and MDC for accuracy, repeatability, and reproducibility are reported in the embedded table.

For volume results (Figure 9), bias ranged between −0.04% and 0.05%, and MDC ranged between 0.21% and 0.49%. Limits of agreement spanned between −0.45% and 0.53% for accuracy, repeatability, and reproducibility. This is well below the maximum of 3.5% considered an acceptable threshold. 

## 4. Discussion

The aim of this work was to assess, for the first time, the use of the EINScan Pro 2X Plus 3D Scanner with HD Prime Pack in digitizing limb models representing different socket shapes in a multi-site setting. High accuracy, repeatability, and reproducibility are needed to ensure that the scanning process captures clinically relevant details across fabrication steps, from before to after rectification, when scans are collected by multiple operators, with physically different 3D scanners of the same model. This study is the first to assess accuracy, repeatability, reproducibility of a 3D scanner considering operators from different sites and distinct 3D scanner units of the same model. It is important to consider this metric when using a 3D scanner across multiple settings, for either research or clinical purposes. 

Data were analyzed using both global and O&P-relevant metrics. Specifically, five outcome measures were analyzed: MRE, MAE, RMSE-RE, and limits of agreement for perimeters and volumes. Results were all well within clinically relevant acceptability thresholds: MRE 0.1 mm (<0.25 mm), MAE 2.5° (<4°), and RMSE-RE 0.52 mm (<1 mm). The limits of agreement for volumes and perimeters were within ±0.53%. 

Our results compare favorably to those reported for other 3D scanners. Seminati et al. [12] studied the accuracy, reproducibility, and repeatability of the Artec Eva 3D Scanner (Artec 3D, Niederanven, Luxembourg), using the Romer Laser 3D Scanner (Hexagon, London, UK) as the gold standard reference. For error in accuracy, an RMSE-RE value of 0.98 mm was reported, which is higher than the error in accuracy reported here. Our results were also lower for the error in accuracy of perimeters, as we could not detect a trend in bias across the sections, and errors in accuracy were lower than 1%. Similar conclusions can be drawn for errors in volume accuracy (bias 1.4%, MDC 1.01%), repeatability (MDC 0.50%), and reproducibility (MDC 0.70%).

Regarding volumes, our results compare favorably with those of Dickinson et al. [14], who reported an accuracy for the VIUScan (Creaform Inc., Levis, QC, Canada) with bias of −0.48% and MDC of 0.24%. Better results were obtained for the limits of agreement of perimeters and volumes, in comparison to reproducibility results reported by Dickinson et al. [15] (with MDC of about 3.5%). However, in this latter study, scans of biological residual limbs were undertaken instead of rigid limb models as in our study, which might explain the difference. 

In this study, we decided to assess the performance of a structured-light 3D scanner at each site instead of a laser 3D scanner because the former does not require the use of adhesive markers attached to the target object for scanning. Structured-light 3D scanners are faster to use and in general, are available at a lower cost. Indeed, the time taken at each site did not exceed a total of five minutes from 3D scanner activation to mesh generation.

It should be noted that this study did not assess performance of the structured-light 3D scanner for directly scanning biological residual limbs. Scanning of limb models allowed us to ensure that the same shapes were scanned at multiple sites, thus controlling for one potentially confounding variable that would have affected the assessment of 3D scanner performance. In this framework, it would have been impractical to move subjects across testing sites. However, scanning limb models likely resulted in better accuracy, repeatability, and reproducibility than would occur when scanning biological limbs given that scanning of biological limbs is more susceptible to motion artifact. A more general limitation for all studies of the performance of 3D scanners is that there is no universally agreed set of outcome measures for comparison. 

Additionally, the identification of anatomical landmarks on OP0 and the alignment of the limbs in the global coordinate system within the software were conducted by agreement of two experienced prosthetists. While differential results among the scans are not affected by this procedure, this limits the assessment of absolute values for volumes and perimeters. Therefore, future research should describe a standardized method for the definition of coordinate systems of residual limbs. The availability of highly accurate, repeatable, and reproducible 3D scanners supports future research in the field of quantitative shape analysis, e.g., to study the evolution of the shape of a residual limb from casting to socket shape or the evolution of the shape of a residual limb over the clinical history of the patient. These techniques can support development of novel AI assisted manufacturing software for prosthetics.

## 5. Conclusions 

The evaluation of the accuracy, repeatability, and reproducibility of 3D digital scanners across multiple sites is a very relevant problem that has never been addressed before in prosthetics. Assessments in this regard are essential before merging results across different sites. The hand-held EINScan Pro 2X Plus 3D Scanner with HD Prime Pack (SHINING 3D Tech. Co., Ltd., Hangzhou, China) was shown to have accuracy, repeatability, and reproducibility well below clinically relevant thresholds. Specifically, the 3D scanner had a MRE of 0.1mm, MAE of 2.5°, and RMSE-RE of 0.52 mm. The limits of agreement for volumes and perimeters were within ±0.53%. Moreover, outcomes were comparable or better than alternative commercially available 3D scanners with performance evaluation reported in the literature. Therefore, results support its use by multiple operators and in multi-site testing in prosthetics. 

## Figures and Tables

**Table 1:** Height and volume of the residual limb models as measured by OP0, in descending order. Both the total height of the residual limb models and the height between the most distal point (A) and the proximal anatomical landmark (midpoint Between the origin of Adductor longus and the ischial Ramus (BAR) for transfemoral (TF) models and Mid Patellar Tendon (MPT) for transtibial (TT) models), are reported.

Model	Total Height [mm]	Height [mm] TF: (A—BAR) TT: (A—MPT)	Volume [dL] TF: (A—BAR) TT: (A—MPT)
TF Ischial	507.2	318.1	33.9
TF Aqua	442.1	269.2	36.3
TT Conical	429.4	213.8	14.7
TT Cylindrical	396.3	195.3	19.5

**Table 2:** Results for the root mean square error (RMSE) of the radial error (RMSE-RE). For each comparison, we report the minimum and maximum values across all four residual limb models and three testing sites. The aligned scans of OP1-A on OP0 were referred to as OP1-A*.

Comparison		Radial Error (RE) [mm]	
		RMSE Min	RMSE Max
Accuracy	OP0 vs. OP1-A	0.06	0.25
	OP0 vs. OP1-B	0.06	0.21
	OP0 vs. OP2	0.06	0.27
Repeatability	OP1-A* vs. OP1-B	0.04	0.16
Reproducibility	OP1-A* vs. OP2	0.04	0.52

## Data Availability

The data presented in this study are openly available in Dryad at [24] https://doi.org/10.5061/dryad.37pvmcvsm (accessed on 26 March 2024).

## Supplementary Materials

The following supporting information can be downloaded at: https://www.mdpi.com/article/10.3390/s24072350/s1, Table S1. Global and O&P Relevant Metrics for metrological scanner and by site.

## Abbreviations

HD	High-Definition
OP0	European independent Professional Operator
OP1	Operator 1, with experience using the 3D scanner
OP1-A	Operator 1, first scan
OP1-A*	the aligned scans of OP1-A on OP0
OP1-B	Operator 1, second scan
OP2	Operator 2, with minimal prior experience using the 3D scanner
VDI/VDE	Association of German Engineers and Association for Electrical, Electronic, and Information Technologies
PETG	Polyethylene Terephthalate Glycol
USA1	First United States site
USA2	Second United States site
EU	European site
O&P	Orthotic and Prosthetic
MRE	mean radial error
*V^R^*	reference mesh
$\vec{d}$	distance between meshes
RE	radial error
*V^M^*	measured mesh
*n_points_*	mesh points
RMSE-RE	root mean square error of the radial distance
MAE	mean angle error
AE	angle error
$\vec{n^{R}}$	normal vector to the reference mesh
$\vec{n^{M}}$	normal vector to the measured mesh
MCID	minimal clinically important difference
A	Most distal point of the model
MPT	Mid Patellar Tendon
BAR	Midpoint Between the origin of Adductor longus and the ischial Ramus
MDC	minimal detectable change
CR	Coefficient of Repeatability
TT	Transtibial
TF	Transfemoral

## References

[1] Vitali A., Togni G., Regazzoni D., Rizzi C., Molinero G. (2021). A virtual environment to evaluate the arm volume for lymphedema affected patients. Comput. Methods Programs Biomed..

[2] Sidles J., Boone D., Harlan J., Burgess E. (1989). Rectification maps: A new method for describing residual limb and socket shapes. J. Prosthet. Orthot..

[3] Lemaire E., Johnson F. (1996). A quantitative method for comparing and evaluating manual prosthetic socket modifications. IEEE Trans. Rehabil. Eng..

[4] Lemaire E., Bexiga P., Johnson F., Solomonidis S., Paul J. (1999). Validation of a quantitative method for defining CAD/CAM socket modifications. Prosthet. Orthot. Int..

[5] Fatone S., Johnson W., Tran L., Tucker K., Mowrer C., Caldwell R. (2017). Quantification of rectifications for the Northwestern University Flexible Sub-Ischial Vacuum Socket. Prosthet. Orthot. Int..

[6] Steer J., Grudniewski P., Browne M., Worsley P., Sobey A., Dickinson A. (2020). Predictive prosthetic socket design: Part 2-generating person-specific candidate designs using multi-objective genetic algorithms. Biomech. Model Mechanobiol..

[7] Steer J., Worsley P., Browne M., Dickinson A. (2020). Predictive prosthetic socket design: Part 1-population-based evaluation of transtibial prosthetic sockets by FEA-driven surrogate modelling. Biomech. Model Mechanobiol..

[8] Dickinson A., Diment L., Morris R., Pearson E., Hannett D., Steer J. (2021). Characterising residual limb morphology and prosthetic socket design based on expert clinician practice. Prosthesis.

[9] Raschke S. (2022). Limb prostheses: Industry 1.0 to 4.0: Perspectives on technological advances in prosthetic care. Front. Rehabil. Sci..

[10] Mayo A., Gould S., Cimino S., Glasford S., Harvey E., Ratto M., Hitzig S. (2022). A qualitative study on stakeholder perceptions of digital prosthetic socket fabrication for transtibial amputations. Prosthet. Orthot. Int..

[11] Redaelli D.F., Barsanti S.G., Biffi E., Storm F.A., Colombo G. (2021). Comparison of geometrical accuracy of active devices for 3D orthopaedic reconstructions. Int. J. Adv. Manuf. Technol..

[12] Seminati E., Canepa Talamas D., Young M., Twiste M., Dhokia V., Bilzon J. (2017). Validity and reliability of a novel 3D scanner for assessment of the shape and volume of amputees’ residual limb models. PLoS ONE.

[13] Seminati E., Young M., Canepa Talamas D., Twiste M., Dhokia V., Bilzon J. (2022). Reliability of three different methods for assessing amputee residuum shape and volume: 3D scanners vs. circumferential measurements. Prosthet. Orthot. Int..

[14] Dickinson A., Steer J., Woods C., Worsley P. (2016). Registering methodology for imaging and analysis of residual-limb shape after transtibial amputation. J. Rehabil. Res. Dev..

[15] Dickinson A., Donovan-Hall M., Kheng S., Bou K., Tech A., Steer J., Metcalf C., Worsley P. (2022). Selecting appropriate 3D scanning technologies for prosthetic socket design and transtibial residual limb shape characterization. J. Prosthet. Orthot..

[16] Kofman R., Beekman A., Emmelot C., Geertzen J., Dijkstra P. (2018). Measurement properties and usability of non-contact scanners for measuring transtibial residual limb volume. Prosthet. Orthot. Int..

[17] Kofman R., Winter R., Emmelot C., Geertzen J., Dijkstra P. (2022). Clinical usability, reliability, and repeatability of noncontact scanners in measuring residual limb volume in persons with transtibial amputation. Prosthet. Orthot. Int..

[18] Hernandez A., Lemaire E. (2017). A smartphone photogrammetry method for digitizing prosthetic socket interiors. Prosthet. Orthot. Int..

[19] Sanders J., Severance M., Allyn K. (2012). Computer-socket manufacturing error: How much before it is clinically apparent?. J. Rehabil. Res. Dev..

[20] Mehmood W., Abd Razak N., Lau M., Chung T., Gholizadeh H., Abu Osman N. (2019). Comparative study of the circumferential and volumetric analysis between conventional casting and three-dimensional scanning methods for transtibial socket: A preliminary study. Proc. Inst. Mech. Eng. Part H.

[21] Bartlett J., Frost C. (2008). Reliability, repeatability and reproducibility: Analysis of measurement errors in continuous variables. Ultrasound Obstet. Gynecol..

[22] Verein Deutscher Ingenieure e.V. VDI/VDE 2634, Optical 3D Measurement Systems—Multiple View Systems Based on Area Scanning. 2008. Engl. VDI/VDE-Gesellschaft Mess- und Automatisierungstechnik: Dusseldorf, Germany. https://www.vdi.de/en/home/vdi-standards/details/vdivde-2634-blatt-3-optical-3d-measuring-systems-multiple-view-systems-based-on-area-scanning.

[23] Cutti A., Osti G., Migliore G., Cardin D., Venturoli F., Verni G. (2018). Clinical Effectiveness of a Novel Hydrostatic Casting Method for Transfemoral Amputees: Results from the First 64 Patients. O&P News.

[24] Fatone S., Cutti A.G. (2024). Limb Model Scans [Dataset].

[25] Cutti A., Fatone S., Hansen A., Group R.L.S.C. (2023). Technique to assess shape captured by two casting approaches. Prosthet. Orthot. Int..

[26] Cignoni P., Callieri M., Corsini M., Dellepiane M., Ganovelli F., Ranzuglia G. (2008). MeshLab: An open-source Mesh processing tool. Computing.

[27] Schroeder W., Martin K., Lorensen B., Kitware Inc. (2006). The Visualization Toolkit: An Object-Oriented Approach to 3D Graphics.

[28] Qt Company Ltd. Qt for Python. https://doc.qt.io/qtforpython-6/index.html#documentation.

[29] Geil M. (2005). Consistency and accuracy of measurement of lower-limb amputee anthropometrics. J. Rehabil. Res. Dev..

[30] Sanders J., Fatone S. (2011). Residual limb volume change: Systematic review of measurement and management. J. Rehabil. Res. Dev..

[31] Bland J., Altman D. (2003). Applying the right statistics: Analyses of measurement studies. Ultrasound Obstet. Gynecol..

[32] Bland M. (2000). An Introduction to Medical Statistics.

[33] Lilja M., Öberg T. (1997). Proper time for definitive transtibial prosthetic fitting. J. Prosthet. Orthot..

[34] Sanders J., Youngblood R., Hafner B., Cagle J., McLean J., Redd C., Dietrich C., Ciol M., Allyn K. (2017). Effects of socket size on metrics of socket fit in trans-tibial prosthesis users. Med. Eng. Phys..

[35] Lilja M., Johansson S., Öberg T. (1999). Relaxed versus activated stump muscles during casting for trans-tibial prostheses. Prosthet. Orthot. Int..

